# Editorial: Insights in psychosomatic medicine: 2023

**DOI:** 10.3389/fpsyt.2025.1571016

**Published:** 2025-07-17

**Authors:** Stephan Zipfel, Rebecca Erschens, Ruth Becker, Katrin Giel

**Affiliations:** ^1^ Department of Psychosomatic Medicine and Psychotherapy, Medical University Hospital Tübingen, Tübingen, Germany; ^2^ German Centre for Mental Health (DZPG), Tübingen, Germany

**Keywords:** planetary health, psychosomatic medicine, global mental health, enhanced psychotherapy, bio-psycho-social model

The theme of the 27th World Congress of the International College of Psychosomatic Medicine (ICPM) was “*Advancing Psychosomatic Medicine in a Challenging World*”. With nearly 500 participants from over 30 countries and 6 continents our event was truly global. Europe and North America were well represented, and Asia, particularly Japan, China and the Republic of Korea, also sent strong delegations. Our scholarship programmes targeted support to colleagues from the global south and, particularly those from crisis regions. We actively encouraged colleagues from low and lower-middle-income countries to participate and were able to involve colleagues from Ukraine (e.g. from the Ivano-Frankivsk National Medical University) in the congress through targeted scholarship programmes.

The ICPM Programme Committee was also determined to actively involve early-career scientists (ECS) in the congress design and programme. We worked closely with the German College of Psychosomatic Medicine (DKPM) and invited the current year of the ‘Clinical Research’ qualification programme to our congress. This was a resounding success, with the ECS playing a leading role in contributing to the programme. Thanks to the financial support of two Foundations for Psychosomatic Medicine, we awarded additional scholarships to young scientists from Germany and abroad, enabling them to travel to and participate in the congress. The programme covered numerous facets of Psychosomatic Medicine, always with a special focus on unique challenges, but of course also on ways and innovations to address these adequately.

The programme included a series of academic events: 15 lectures, 31 symposia, 11 short lecture sessions, 7 masterclasses and 2 poster sessions with 100 posters presented and discussed by participants from a global bio-psycho-social community (10). The masterclasses were an important contribution to the congress, which was especially appreciated by the ECS. In this context, close contact between experienced researchers and clinicians and young colleagues is particularly helpful for the transfer of knowledge and for career coaching. We presented the special features of the well-developed discipline of Psychosomatic Medicine in Germany by including the German College of Psychosomatic Medicine, which was founded in 1974 and has celebrated its 50th birthday in the congress year 2024. For over 25 years, there has been an independent specialist and psychosomatic medical association (DGPM) in Germany, with more than 280 psychosomatic hospitals, independent psychosomatic departments and more than 4120 practising psychosomatic specialists (1). The establishment of the German Centre for Mental Health (DZPG) is now promoting research into and improvement of the translational care for people with mental conditions in Germany (2). The Tübingen site of the DZPG has therefore also supported the ICPM congress.

We contrasted this with the sometimes very difficult care situation in other countries, inviting prominent researchers in our field and related disciplines to present their questions and findings in a series of lectures. The congress programme reflected the tension between global developments, challenges and national responses. Vikram Patel from Harvard, United States (3) – who published the key points of the Global Mental Health Initiative with the Lancet Commission in 2023 – was one of the keynote speakers. He spoke about the importance of psychosocial interventions for global mental health, and another important Research Topic was psychosomatic and mental illnesses that develop over the course of a lifetime. We were also honoured to welcome Pat McGorry from Melbourne, Australia (4), who published the Lancet Psychiatry Commission on youth mental health at the beginning of September 2024. The development of psychotherapy has clearly brought us visible progress in the treatment of a wide range of mental and psychosomatic illnesses in recent years. Emily Holmes (University of Uppsala, Sweden), who led the Lancet Commission on Psychotherapy (5), provided groundbreaking impetus, especially in the treatment of people with trauma. Giovanni Fava (Buffalo, United States) was awarded the first ever ICPM Lifetime Achievement Award in recognition of his important contribution to the implementation of the biopsychosocial concept in clinical work and the development of Well Being Therapy (6).

The challenge of climate change necessitates profound and rapid changes in all areas of society. Achieving global sustainability goals requires technical innovations and political measures, along with changes in people’s everyday behaviour in areas such as energy consumption, mobility and patterns of consumption. We addressed the issue of climate change and its impact on physical and mental health, placing a strong emphasis on “Planetary health and social transformation concerning climate issues” with a Key Lecture by Christina Demski (University of Bath, UK), and other international experts also contributed to this Research Topic.

However, we also looked at current topics in our field, such as the possibilities of using enhanced psychotherapy to treat patients with psychosomatic illnesses even more successfully (6). This also included recent study results on the health issues of SARS-CoV-2 (7), psycho-oncology and low-threshold interventions in somatic clinics (8).

In addition, the discourse centred around the utilisation of digital tools and artificial intelligence to enhance the targeted and effective treatment of patients with mental conditions. The promotion of interprofessional communication and the development of professional identities in student education (Erschens et al.) were also explored, particularly for future psychosomatic doctors and other healthcare professionals.

Overall, it was a great pleasure and honour to host the ICPM Congress in Tübingen for the second time in Germany. As this congress is held every two years in a different country and in the majority of cases even on a different continent, it is truly international in its character and offers valuable opportunities to meet scientists and clinicians from all over the world. Psychosomatic Medicine is embedded in the health care system in very different ways internationally, so the discussions and exchange of ideas were very lively and broadening the horizons (9).
